# Predicting immune risk in treatment-naïve HIV patients using a machine learning algorithm: a decision tree algorithm based on micronutrients and inversion of the CD4/CD8 ratio

**DOI:** 10.3389/fnut.2024.1443076

**Published:** 2024-10-16

**Authors:** Saurav Nayak, Arvind Singh, Manaswini Mangaraj, Gautom Kumar Saharia

**Affiliations:** ^1^Department of Biochemistry, All India Institute of Medical Sciences (AIIMS), Bhubaneswar, Bhubaneswar, India; ^2^Department of Community Medicine and Family Medicine, All India Institute of Medical Sciences (AIIMS), Bhubaneswar, Bhubaneswar, India

**Keywords:** HIV, micronutrients, machine learning and AI, CD4/CD8 lymphocytes + +, treatment naive patients, zinc, calcium, magnesium

## Abstract

**Introduction:**

Micronutrients have significant functional implications for the human immune response, and the quality of food is a major factor affecting the severity and mortality caused by HIV in individuals undergoing antiretroviral therapy. A decrease in CD4 lymphocyte count and an increase in CD8 lymphocyte count are the hallmarks of HIV infection, which causes the CD4/CD8 ratio to invert from a normal value of >1.6 to <1.0. In this study, we tried to analyze whether the nutritional status of HIV-positive patients has an impact on the CD4/CD8 ratio inversion by utilizing a machine learning (ML) algorithm.

**Methods:**

In this study, 55 confirmed HIV-positive patients who had not started their anti-retroviral therapy were included after obtaining their informed, written consent. Moreover, 55 age-and sex-matched relatives and caregivers of the patients who tested negative in the screening were enrolled as controls. All individual patient data points were analyzed for model development with an 80–20 train–test split. Four trace elements, zinc (Zn), phosphate (P), magnesium (Mg), and calcium (Ca), were utilized by implementing a random forest classifier. The target of the study was the inverted CD4/CD8 ratio.

**Results:**

The data of 110 participants were included in the analysis. The algorithm thus generated had a sensitivity of 80% and a specificity of 83%, with a likelihood ratio (LR+) of 4.8 and LR-of 0.24. The utilization of the ML algorithm adds to the limited evidence that currently exists regarding the role of micronutrients, especially trace elements, in the causation of immune risk. Our inherent strength lies in the fact that this study is one of the first studies to utilize an ML-based decision tree algorithm to classify immune risk in HIV patients.

**Conclusion:**

Our study uniquely corroborated the nutritional data to the immune risk in treatment-naïve HIV patients through the utilization of a decision tree ML algorithm. This could subsequently be an important classification and prognostic tool in the hands of clinicians.

## Introduction

HIV/AIDS (human immunodeficiency virus/acquired immunodeficiency syndrome) is a global pandemic. The first case of HIV infection was reported in India in 1986, and since then, the disease has spread throughout the country. The country’s estimated 2,349,000 HIV/AIDS-positive citizens had an adult prevalence of 0.22% in 2019. Moreover, 3.4% of all people living with HIV/AIDS (PLWHA) were reported to be children. Approximately 44% of the PLWHA aged 15 years and older were reported to be female patients (1). Higher rates of HIV infection, viral load, and sexual and vertical transmission of the virus are all significantly more prevalent in developing nations, such as India, due to malnutrition and food insecurity (2). HIV is characterized by the suppression of the immune system, which increases the energy demands of the already emaciated HIV patients in the fight against the infection and triggers greater nutritional complications (3).

Micronutrients have significant functional implications for the human immune response, and the quality of food is a major factor affecting the severity and mortality caused by HIV in individuals undergoing antiretroviral therapy (4). When highly active antiretroviral therapy (HAART) was not available, several studies involving HIV-positive adults demonstrated that multivitamin supplementation improved overall clinical outcomes, decreased viral load, improved immunological reconstitution, and decreased mortality (5). Adequate calories from macronutrients and micronutrients constitute an acceptable diet, which is necessary to improve immunity to fight infection, sustain patients’ nutritional status, reduce the spread of HIV, and enhance quality of life (6). To improve nutrient intake and diminish viral load by boosting immunity, dietary treatments such as macronutrient and micronutrient supplements, nutrition education or counseling, and food assistance programs are crucial for HIV-positive individuals (7).

A decrease in CD4 lymphocyte count and an increase in CD8 lymphocyte count are the hallmarks of HIV infection, which causes the CD4/CD8 ratio to invert from a normal value of more than 1.6 to <1.0. It has long been known that the CD4/CD8 ratio can be used to predict the progression of HIV infection (8). Therefore, in this study, we tried to analyze whether the nutritional status of HIV-positive patients has an impact on the CD4/CD8 ratio inversion by utilizing a machine learning (ML) algorithm. This will allow us to specifically generate hierarchical rules for the early prediction of increased immune risk in treatment-naïve HIV patients based on these micronutrients.

## Materials and methods

### Study design and setting

This study was a case–control study conducted in the Department of Biochemistry at a tertiary care institute in eastern India. Ethical clearance was obtained from the Institutional Ethics Committee with reference no. T/IM-F/18–19/07 dated 15 May 2023.

### Selection of participants

A total of 55 patients over 18 years of age attending the Integrated Counselling and Testing Centre (ICTC) clinic, were included. Pre-operative patients, clinically symptomatic patients referred by the clinician, and normal or high-risk patients who directly walked into the clinic for HIV testing with a positive screening test (MeriScreen) and a confirmatory test for HIV (HIV TRI-DOT test) and who had not started anti-retroviral therapy were included in this study, after obtaining written, informed consent from them. Patients who were seriously ill, pregnant women, those taking vitamin supplements, and those unable to answer the questions were excluded from the study. Furthermore, 55 age- and sex-matched relatives and caregivers of the patients, who were matched by age and sex and tested negative on screening, were enrolled as controls after obtaining written informed consent from them.

### Procedure

A detailed dietary history of the patients, with the frequency of non-vegetarian foods and drug intake, was recorded on their datasheets. Anthropometric measurements, such as height, weight, and body mass index (BMI), were recorded for both the patients and controls. A clinical examination of the patients was also performed before blood collection. A total of 5 mL of blood—2 mL in an ethylenediaminetetraacetic acid (EDTA) vial and 3 mL in a plain vial—was collected from both patients and controls at the ICTC clinic itself. The CD4+ and CD8+ cell counts were analyzed using a Beckman Coulter Navios Flow cytometer (Beckman Coulter Ireland, Inc., Lismeehan). The total concentration of calcium (Ca), magnesium (Mg), phosphate (P), and zinc (Zn) in the serum was estimated using a Beckman Coulter AU480 Autoanalyzer. For zinc concentration, Randox quality control material was used and for the rest of the biochemical parameters, Bio-Rad internal quality control samples were used daily.

### Statistical analysis

Statistical analysis was performed using SPSS v26. The data were expressed as mean ± standard deviation. Independent samples *t*-test was performed to compare groups, and correlation was performed using Pearson’s correlation coefficient. A *p* < 0.05 was considered statistically significant.

### Machine learning modeling

The data were entered into MS Excel for modeling. All machine learning-related analyses were performed using Python 3.6 in a Jupyter Notebook. All individual patient data points were analyzed for model development with an 80–20 train–test split. In the training dataset, the four micronutrients, i.e., Zn, P, Ca, and Mg, were processed using a custom sequential permutation model based on a random forest classifier model (minimum split = 100, criterion = Gini, and max. Depth = none), with the inverted CD4/CD8 ratio as the target. Each of the iterative classifiers was evaluated for accuracy, recall, precision, F1 value, Matthews correlation coefficient (MCC), and area under the curve (AUC) for the test parameters. Using the means of these matrices, the overall optimized score (OOS) was calculated. The parameter or combination of the parameters with the highest OOS value was selected as the most optimal combination. To generate a clinical decision algorithm, this selected combination was applied to a decision tree model (based on classification and regression tree (CART) with criterion = Gini, splitter = best, max. Depth = 5, and min. Sample splits = 2). This model was validated by a 10-fold cross-validation (scoring = F1 score). The model predictions were evaluated by area under the curve (AUC), sensitivity, specificity, and likelihood ratio (LR).

## Results

The data from 110 participants in total were included in the analysis. The demographic and biochemical data of the population are tabulated in Table 1.

**Table 1:** Characteristics of the study population with a comparison between the groups.

		Controls	Cases	*p*-value^#^
Sex	Men	42 (76.3)	42 (76.3)	1*^χ^*
	Women	13 (23.7)	13 (23.7)	
Age		37.1 ± 12	37.1 ± 11.9	0.994
CD4 count		965.1 ± 294.9	250.7 ± 195.1	<0.001
CD8 count		566.6 ± 212.7	837.9 ± 680.1	0.006
CD4/CD8 ratio		1.8 ± 0.6	0.3 ± 0.2	<0.001
Calcium		9.3 ± 1.8	7.9 ± 2.3	<0.001
Phosphorus		4.6 ± 1.9	3.2 ± 1.2	<0.001
Magnesium		1.5 ± 0.5	1.6 ± 0.5	<0.001
Zinc		57.2 ± 19.6	28.1 ± 16.8	<0.001
Education status	Educated	51 (92.5)	1 (1.9)	<0.001 ^χ^
	Uneducated	4 (7.5)	54 (98.1)	
Occupational status	Employed	52 (95)	1 (1.9)	<0.001 ^χ^
	Unemployed	3 (5)	54 (98.1)	
Dietary history	Vegetarian	3 (5)	7 (12.5)	0.321 ^χ^
	Non-vegetarian	52 (95)	48 (87.5)	

^#Independent samples t-test. χChi-squared test.^

**Figure 1:**
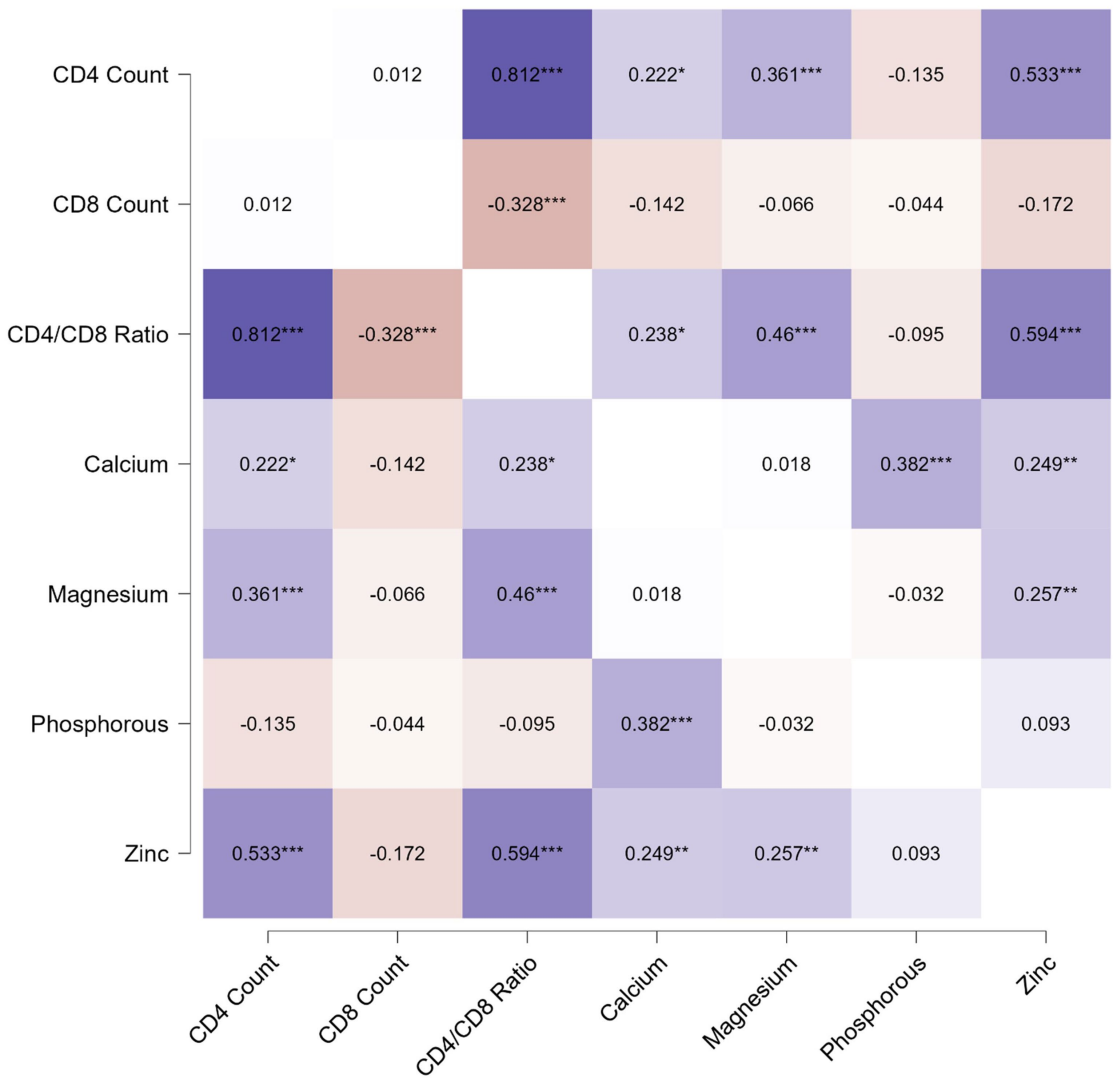
Pearson correlation heatmap for analysis between immunological parameters and micronutrients.

The correlation analysis showed a significant positive correlation of calcium, magnesium, and zinc with the CD4 count and CD4/CD8 ratio. The Pearson’s correlation coefficients and *p*-value are tabulated in Table 2. The correlation heatmap is shown in Figure 1.

**Table 2:** Pearson’s correlation coefficients of micronutrients with immunological parameters.

	CD4 count*	CD8 count*	CD4/CD8 ratio*
Calcium	0.222 (0.020)	−0.142 (0.139)	0.238 (0.012)
Phosphorus	−0.135 (0.158)	−0.066 (0.495)	−0.095 (0.323)
Magnesium	0.361 (<0.001)	−0.044 (0.651)	0.460 (<0.001)
Zinc	0.533 (<0.001)	−0.172 (0.072)	0.594 (<0.001)

*Correlation coefficient (*p*-value).

The evaluation metrics of the test data are tabulated in Table 3.

**Table 3:** Feature evaluation metrics for testing the dataset of various feature combinations along with the overall optimized score (OOS) in descending order.

Features	Testing data						
	Accuracy	Recall	Precision	F1	MCC	AUC	OOS
Ca + Mg + Zn	0.857	0.929	0.813	0.867	0.722	0.857	0.841
Ca + P + Mg + Zn	0.821	0.857	0.800	0.828	0.645	0.821	0.795
P + Mg + Zn	0.786	0.857	0.750	0.800	0.577	0.786	0.759
Ca + P	0.786	0.786	0.786	0.786	0.571	0.786	0.750
Mg + Zn	0.750	0.857	0.706	0.774	0.512	0.750	0.725
Ca + P + Mg	0.750	0.643	0.818	0.720	0.512	0.750	0.699
Ca + P + Zn	0.714	0.857	0.667	0.750	0.447	0.714	0.692
Zn	0.714	0.786	0.688	0.733	0.433	0.707	0.677
Mg	0.714	0.714	0.714	0.714	0.429	0.747	0.672
Ca + Zn	0.679	0.786	0.647	0.710	0.366	0.679	0.644
P + Zn	0.679	0.714	0.667	0.690	0.358	0.679	0.631
Ca + Mg	0.679	0.571	0.727	0.640	0.366	0.679	0.610
P + Mg	0.571	0.643	0.563	0.600	0.144	0.571	0.515
P	0.536	0.571	0.533	0.552	0.072	0.538	0.467
Ca	0.357	0.500	0.389	0.438	−0.298	0.347	0.289

**Figure 2:**
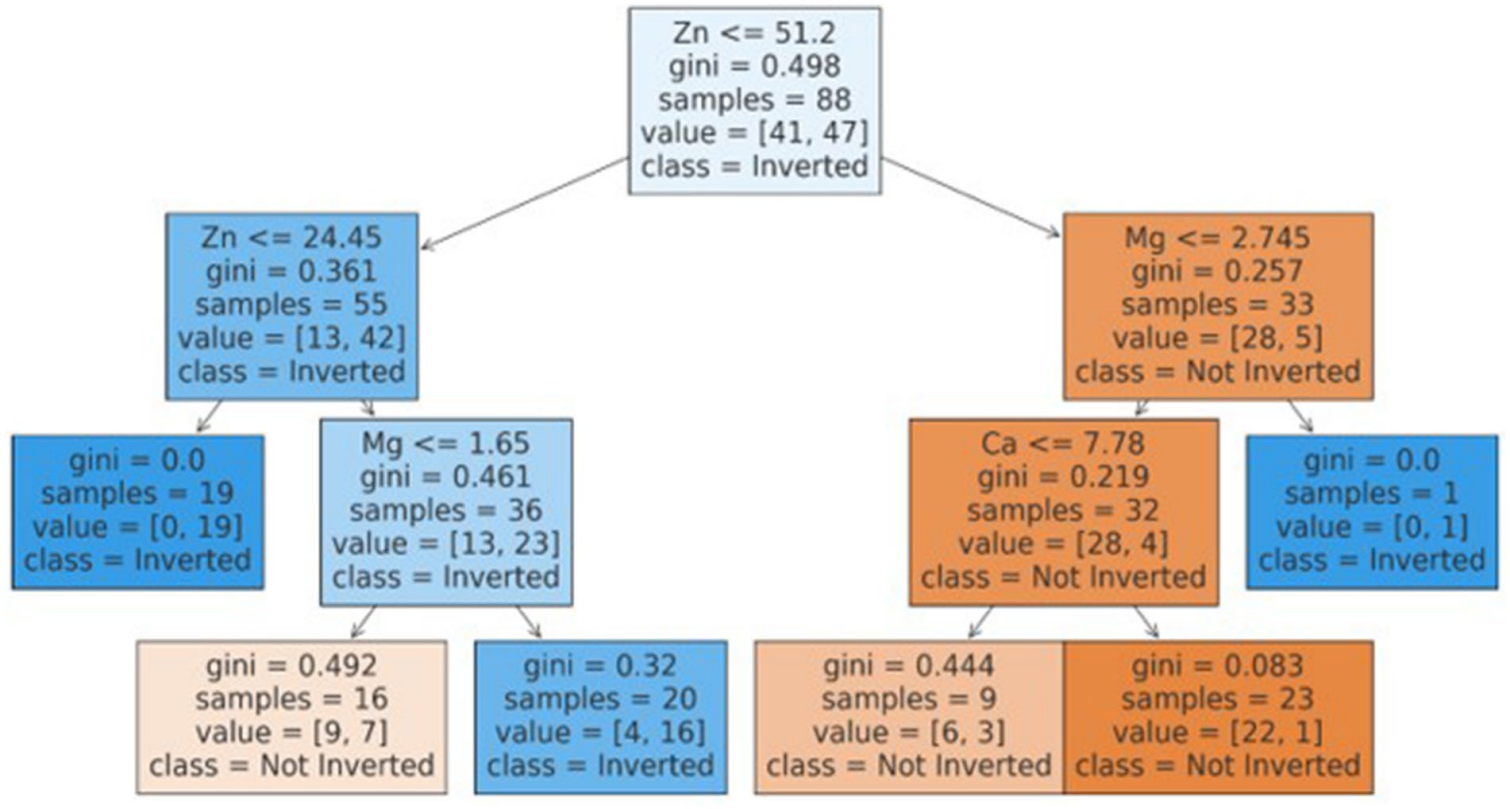
CART based decision tree for classification of inverted ratio.

**Figure 3:**
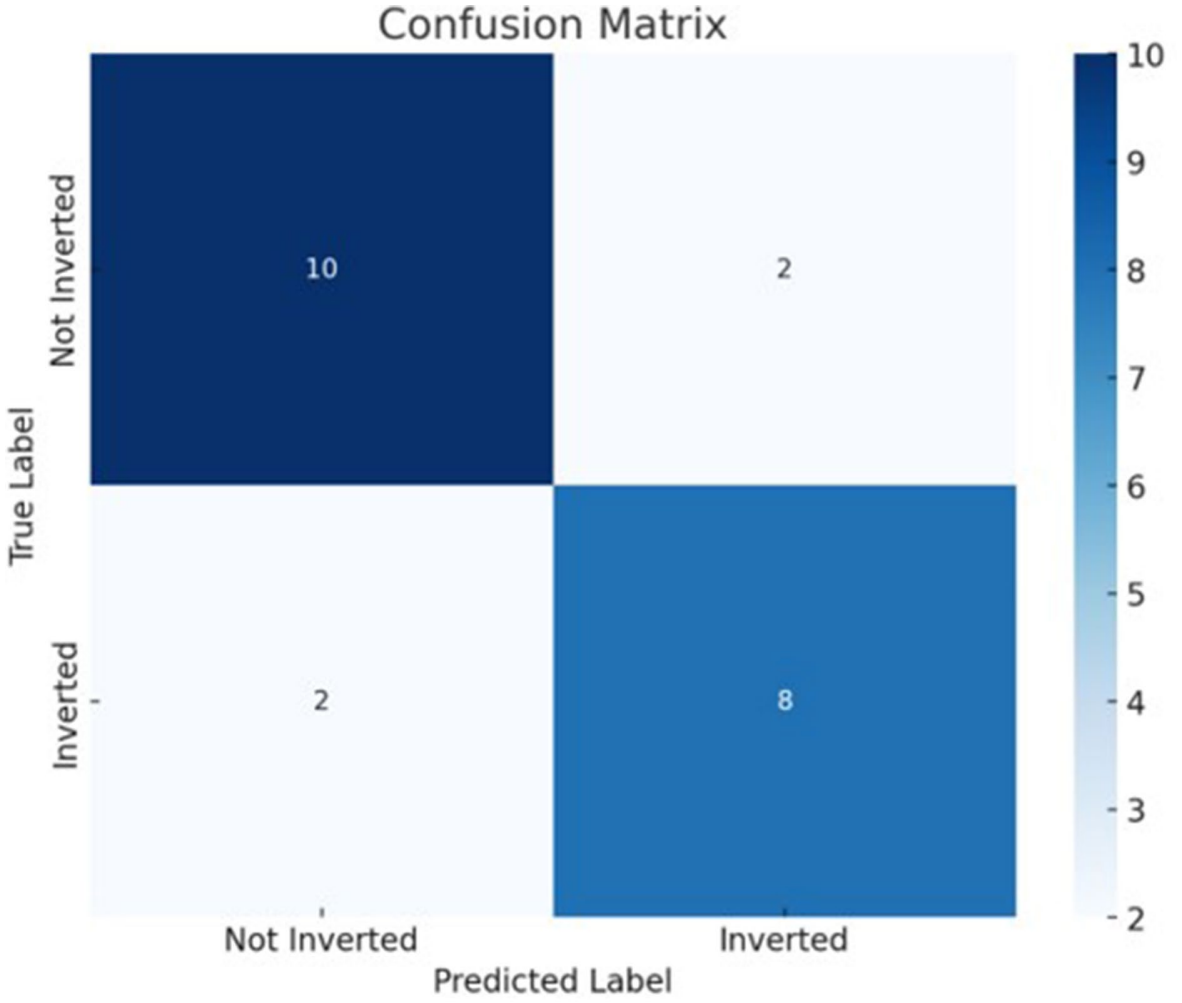
Decision tree based classification confusion matrix.

The best model that included calcium, magnesium, and zinc was implemented to generate a decision algorithm. The algorithm thus generated had an AUC of 0.880, sensitivity of 80%, and specificity of 83%, with LR+ of 4.8 and LR-of 0.24. The diagnostic odds ratio was 20. The mean 10-fold cross-validation was 0.753. The algorithm tree and the confusion matrix are shown in Figures 2, 3, respectively.

Based on the decision tree algorithm, a simplified textual classification was also generated.

ALGORITHM: Our inherent strength lies in the fact that this study is one of the first studies to utilize CART based decision tree for classification of inverted ratio of immune risk in HIV patients.

- If Zn is ≤51.20:

o If Zn is ≤24.45, classify as Inverted (class 1).
o If Zn is >24.45:

▪ If Mg is ≤1.65, classify as Not Inverted (class 0).
▪ If Mg is >1.65, classify as Inverted (class 1).

- If Zn is >51.20:

o If Mg is ≤2.74:

▪ If Ca is ≤7.78, classify as Not Inverted (class 0).
▪ If Ca is >7.78, classify as Not Inverted (class 0).

o If Mg is >2.74, classify as Inverted (class 1).

## Discussion

Our study is one of the pioneering studies in which it was attempted to achieve predictive classification potential regarding the risk of immunocompromised status in treatment-naïve HIV patients. The utilization of an ML algorithm adds to the limited evidence that currently exists regarding the role of micronutrients, especially trace elements, in the causation of immune risk.

CD8 cell proliferation in the general population indicates increased immunosenescence and independently predicts mortality, particularly in older individuals. Individuals with a CD4/CD8 ratio inversion (<1) have CD8 cells with a shorter telomere length, exhibit expression of senescence markers such as CD28-, experience oligoclonal expansion of cytomegalovirus (CMV)-specific CD8+ T cells, and present with impaired thymic function (9, 10). Later investigations on antiretroviral therapy (ART) have revealed that low CD4/CD8 ratios are also associated with underlying inflammation, oxidative stress, decreased thymic production, and poor control of latent viruses such as CMV, Epstein–Barr virus (EBV), or active coinfections such as hepatitis C (9). Importantly, patients with HIV with low CD4/CD8 ratios have higher levels of inflammation and immunosenescence despite receiving otherwise effective treatment (11).

In previous studies, a decrease in serum zinc levels has been implicated in the micronutrient panel of HIV patients. These studies implied that reduced zinc status is generally found in HIV patients with a higher risk of immune-risk linked to the CD+ cell count (5, 12, 13). Zinc has been primarily linked with antiviral immune responses through the mechanisms of antiviral immune signaling pathways (14). Ionic zinc possesses unique and distinct antiviral properties via an immune response led by interferons (IFNs) and is invariably required to clear infections. Vatsalya et al. (15) reported the involvement of decreased zinc levels in T cell apoptosis in an HIV-infected human T cell line, which may contribute to the depletion of CD4+ T cells. In addition, zinc has been shown to contribute to a number of innate and adaptive immune signaling pathways (16). Zinc supplementation has been linked with an increase in cell-mediated markers of immunocompetence, including the number of circulating T lymphocytes, particularly the percentage of CD4+ cells and the CD4+/CD8+ ratio (17). In our study, the algorithm also correlated the propensity of lower zinc levels with a higher probability of predicting increased immune risk in the patients.

As a second intracellular messenger, calcium participates in an array of physiological and biochemical processes, including immune response, utilization of energy, cell division and proliferation, information transfer, and cell development. Furthermore, its signaling offers a target for the invasion, replication, spread, and release of viruses (18, 19). Once the host cell is taken over, viruses use calcium signaling to control apoptosis and hinder host defenses to create a persistent infection (20, 21).

It has been shown that magnesium has potent anti-inflammatory effects as low magnesium levels are linked to increased inflammation and higher magnesium levels have been linked to a decrease in C-reactive protein (CRP) levels (22). The capability of magnesium to activate vitamin D is one of its additional critical roles in the body. Magnesium functions as a cofactor in the enzymes that convert vitamin D from its inactive state to an active state, thereby indirectly correlating with the ability of vitamin D to enhance immune function (23). This was demonstrated in our algorithm, which showed that a lower calcium and magnesium status was associated with an increased likelihood of an inverted CD4/CD8 ratio, consequently indicating immune risk in the patients.

Our inherent strength lies in the fact that this study is one of the first studies in India, and worldwide, to utilize an ML-based decision tree algorithm to classify immune risk in HIV patients. In addition, the innate importance of trace elements, which is frequently overlooked in clinical practice, has been quite evidently demonstrated.

However, this study has a few limitations, primarily the small sample size. Furthermore, there is a need to delve deeper into the nutritional, anthropometric, and biochemical aspects of HIV+ patients.

## Conclusion

Our study uniquely corroborated nutritional data to immune risk in treatment-naïve HIV patients through the utilization of a decision tree ML algorithm. The findings of this study suggest that this algorithm will subsequently be an important classification and prognostic tool for clinicians while treating people with HIV. Similar studies in a multicenter setting with a larger set of data points and more comprehensive markers of an individual’s nutritional status will further validate the results of this study and increase their robustness.

## Data Availability

The original contributions presented in the study are included in the article/supplementary material, further inquiries can be directed to the corresponding author/s.
